# Advances in the Role of Endothelial Cells in Cerebral Small Vessel Disease

**DOI:** 10.3389/fneur.2022.861714

**Published:** 2022-04-11

**Authors:** Tao Bai, Shijia Yu, Juan Feng

**Affiliations:** Department of Neurology, Shengjing Hospital of China Medical University, China Medical University, Shen yang, China

**Keywords:** cerebral small vessel disease (CSVD), endothelial cells (ECs), hypertension, blood-brain barrier, cognitive impairment, white matter hyperintensities (WMH)

## Abstract

Cerebral small vessel disease (CSVD) poses a serious socio-economic burden due to its high prevalence and severe impact on the quality of life of elderly patients. Pathological changes in CSVD mainly influence small cerebral arteries, microarteries, capillaries, and small veins, which are usually caused by multiple vascular risk factors. CSVD is often identified on brain magnetic resonance imaging (MRI) by recent small subcortical infarcts, white matter hyperintensities, lacune, cerebral microbleeds (CMBs), enlarged perivascular spaces (ePVSs), and brain atrophy. Endothelial cell (EC) dysfunction is earlier than clinical symptoms. Immune activation, inflammation, and oxidative stress may be potential mechanisms of EC injury. ECs of the blood–brain–barrier (BBB) are the most important part of the neurovascular unit (NVU) that ensures constant blood flow to the brain. Impaired cerebral vascular autoregulation and disrupted BBB cause cumulative brain damage. This review will focus on the role of EC injury in CSVD. Furthermore, several specific biomarkers will be discussed, which may be useful for us to assess the endothelial dysfunction and explore new therapeutic directions.

## Introduction

Cerebrovascular disease, one of the most important causes of neurological dysfunction, has become an urgent human health issue (1). Cerebral small vessel disease (CSVD) affects almost all people over 90, which is responsible for 45% of cases of dementia in the world (2). CSVD is caused by a disorder in perforating cerebral vessels, and most of the lesions are in cerebral white matter and deep gray matter (3). There is considerable evidence that vascular dysfunction is a fundamental change in CSVD. Vascular endothelial cells (ECs) form the luminal surface of all blood vessels and play an important role in maintaining vascular morphology and biological function (4). Recently, endothelial dysfunction has been considered as a key in the pathogenesis of CSVD and vascular dementia (VD). We focus on the decrease of cerebral blood flow (CBF) and the disruption of blood–brain barrier (BBB) during CSVD. Until now, there is a lack of effective prevention and treatment measures. A better comprehension of pathological mechanisms is beneficial for the investigation of diagnostic biomarkers and the development of treatment targets.

### Arteriosclerosis-Related CSVD

Currently, CSVD is classified into six types according to its etiology (5): arteriosclerosis-related CSVD, amyloid-related CSVD, genetic CSVD (distinct from amyloid angiopathy), inflammatory/immunologically mediated CSVD, venous collagenosis, and other CSVDs. From the pathological point of view, nongenetic CSVD is mainly divided into arteriosclerosis-related CSVD and amyloid-related CSVD (6), and the former CSVD is discussed in our study. However, for brevity, we will simply refer to it as CSVD. The aging of the population is closely related to the occurrence of CSVD, but the contribution of ethnicity in CSVD needs to be further confirmed by epidemiological studies (7, 8). CSVD increases the risk of acute stroke more than 2-fold (9), and its classical form is characterized by cognitive impairment or dementia, motor dysfunction, and psychobehavioral abnormalities. In addition, a typical pattern of cognitive impairment due to CSVD is an impaired executive function with the preservation of memory (5). In the acute phase, its clinical feature is acute stroke syndrome, including hemorrhage and infarction (10).

**Figure 1:**
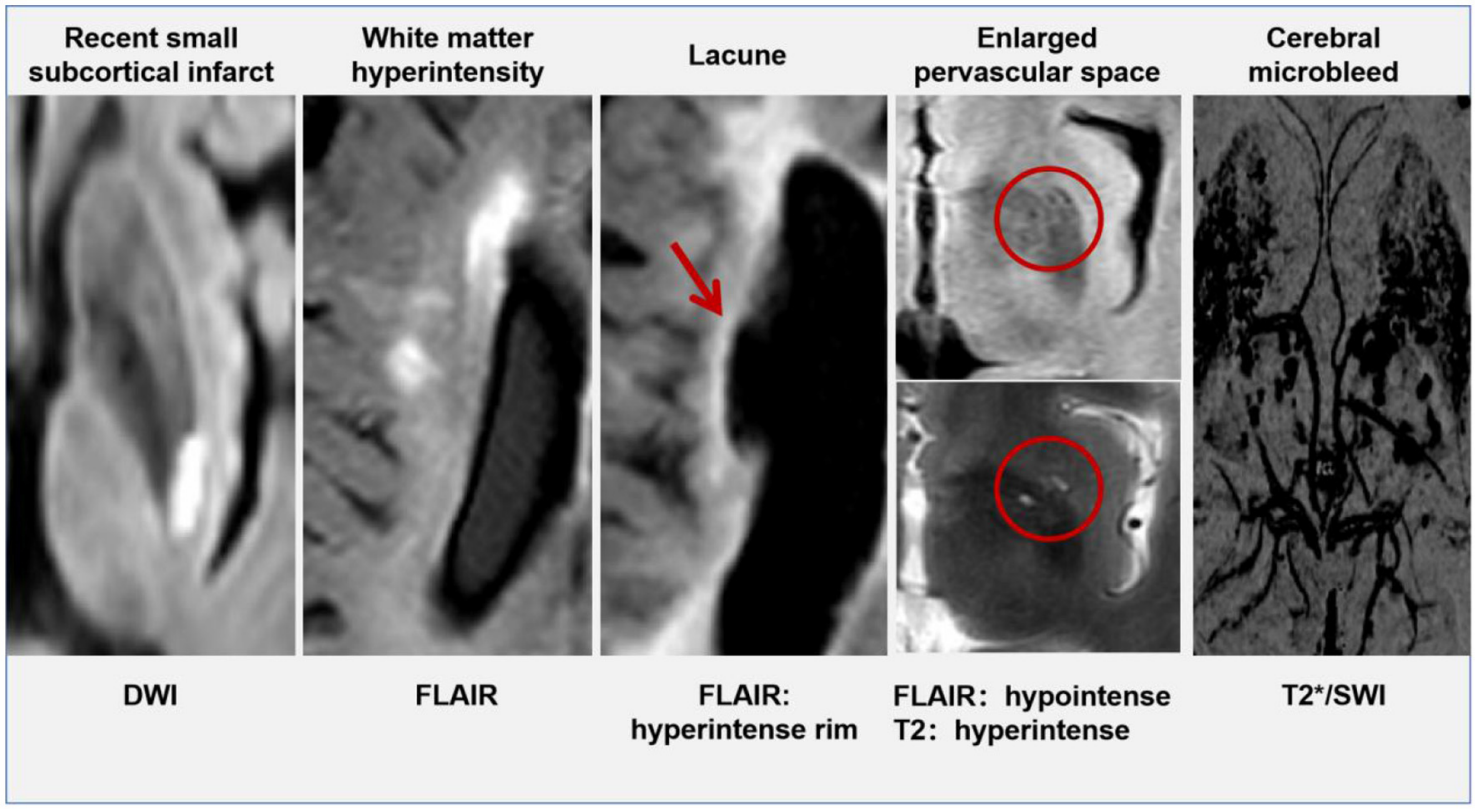
The typical manifestation of cerebral small vessel disease (CSVD) on MRI.

Currently, the opinion that CSVD is a local manifestation of systemic small vessel lesions in the brain is widely accepted (11), scholars have found that patients with CSVD were often accompanied by small vessel damage in other organs, including the kidney and retina (12, 13). The arterial walls demonstrated hyaline degeneration, leading to thickening and narrowing of the arteries eventually. Cerebral small vessel mainly involves the penetrating vessels <1 mm in diameter, including small arteries, micro-arteries, capillaries, and small veins (14). Typical lesions of CSVD were located in the thalamocortical loop and the corticospinal tracts, affecting the information-processing efficiency. Intracranial vessel wall lesions can be identified by 7T magnetic resonance imaging (MRI), but this technique is not yet widely available in clinical centers (15). Advances in imaging help us to check for indirect signs of CSVD on brain MRI. There are six categories of specific changes, including (16) recent small subcortical infarcts, white matter hyperintensity (WMH), lacune, cerebral microbleeds (CMB), enlarged perivascular spaces (ePVS), and brain atrophy. All of these changes may be the result of vascular dysfunction and vascular pathology. The MRI signal characteristics and typical manifestations of CSVD have been shown in Table 1 and Figure 1 (except brain atrophy).

**Table 1:** Magnetic resonance imaging (MRI) characteristics related to cerebral small vessel disease (CSVD).

	**Recent subcortical infarct**	**WMH**	**Lacune**	**ePVS**	**CMB**
T1	↓	—/↓	↓	↓	—
T2	↑	↑	↑	↑	—
DWI	↑	—	—/↓	—	—
FLAIR	↑	↑	↓	↓	—
T2*- weighted GRE	—	↑	—(↓ if haemorrhage)	—	↓(SWI)
diameter	≤ 20mm	—	3−15mm	≤ 2mm	≤ 10mm

*↑ : increased signal; —: iso-intense signal; ↓ : decreased signal*.

### Endothelial Cells

Vascular endothelium, a monolayer of ECs lining the interior walls of vessels, is an important tissue that regulates hemodynamic stability. ECs maintain the balance between coagulation and fibrinolysis and participate in vascular injury, inflammation, and repair (17). First, ECs are able to respond to hemodynamic changes *via* the release of vasoactive substances. For example, when shear stress increases, ECs release nitric oxide (NO), which mediates smooth muscle relaxation in blood vessels. This process begins with the upregulation of NO synthase in ECs (18). Second, ECs can secrete antiplatelet agents, including prostacyclin (PGI) and NO, which prevent platelet aggregation *via* increasing the cyclic adenosine monophosphate (cAMP) content in platelets (19). In addition, ECs can inactivate the clotting factors VIIIa and Va and suppress thrombosis with activation of the protein C/protein S pathway (20). Under the physiological state, ECs exhibit anticoagulant activities. Third, ECs express several innate immune receptors, including the toll-like receptor (TLR) family. When agonists bind to these receptors, the structure of adhesion molecules in ECs will change. This will increase vascular permeability, promote the production of inflammatory cytokines, recruit leukocytes, and reach a procoagulant state (21). Furthermore, ECs also play an important role in the process called angiogenesis, a physiological process by which new blood vessels grow from existing ones. During angiogenesis, activated ECs migrate toward the gradient of vascular endothelial growth factor (VEGF) under hypoxic conditions (22).

**Figure 2:**
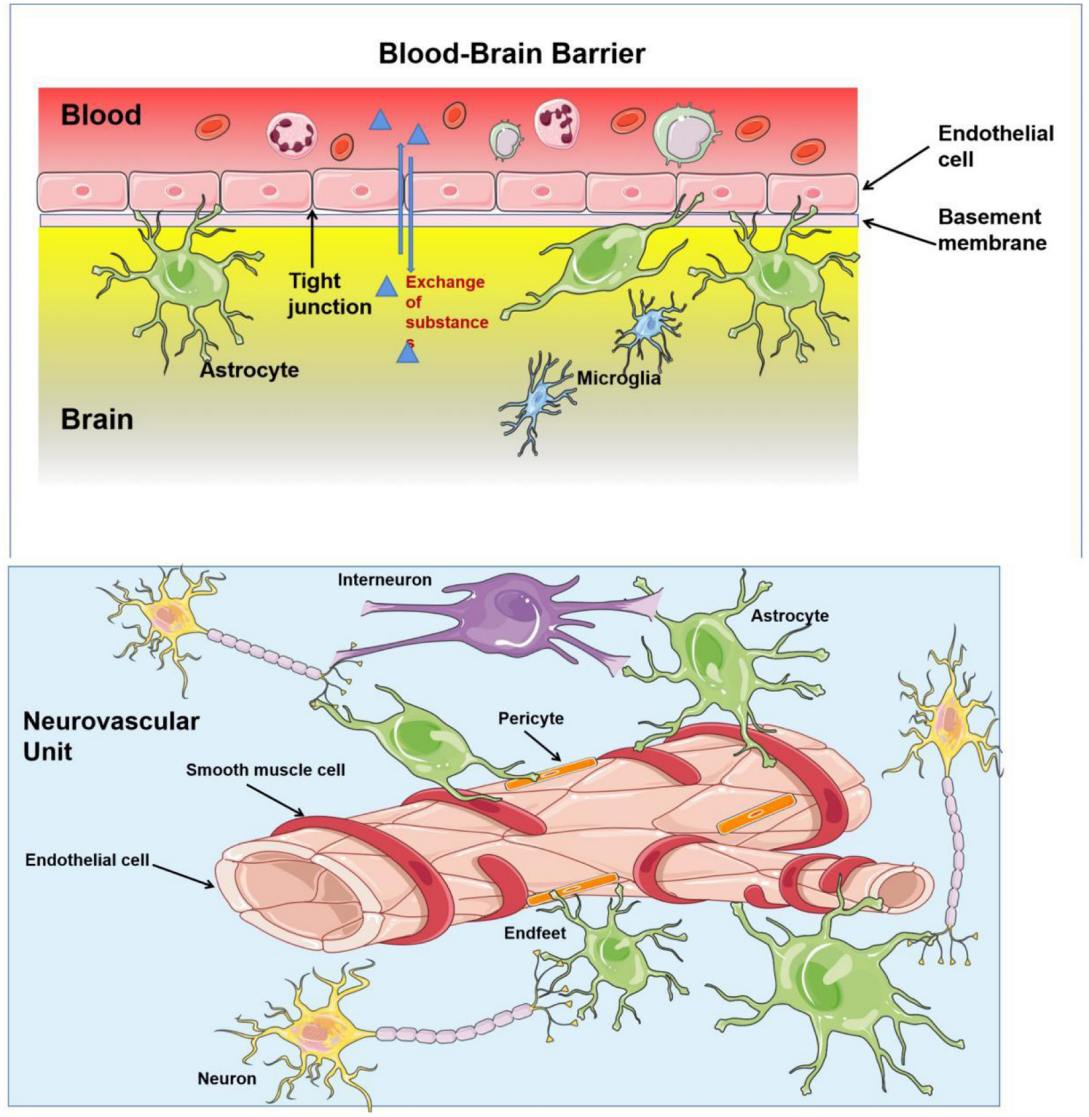
The structure of blood–brain barrier (BBB) and neurovascular unit (NVU). BBB regulates substance influx and efflux to ensure a homeostatic environment for the brain function, which is composed by basement membrane, astrocytes, and endothelial cells (ECs) anchored to each others by tight junction. Neurones, interneurones, astrocytes, smooth muscular cells, pericytes, and ECs are important constituents of NVU, which provides a basic structure for NVC and regulates the cerebral blood flow (CBF).

In the central nervous system, ECs mainly compose the structure of the neurovascular unit (NVU) and BBB (Figure 2). Nutrients transported *via* the blood supply ensure brain activities. However, researchers described for the first time that neuronal structures could influence brain blood flow (23), which regulated the supply of oxygen and nutrients (24). The NVU, a structure composed of neurons, interneurons, astrocytes, basal lamina covered with smooth muscle cells and pericytes, ECs, and an extracellular matrix, ensures the coupling relation between blood supply and neuronal demand (25, 26). ECs can interact with astrocytes and produce vasoactive factors (such as NO) to regulate vascular tone (27). Specifically, autoregulation maintains a nearly constant blood flow to the brain within the range of 50–160 mmHg (28), and hyperemia improves regional CBF by adjusting the changes in the activity of specific brain sectors (29), the phenomenon is called neurovascular coupling (NVC). In addition, complete BBB is the most important factor in maintaining brain tissue homeostasis, which prevents the entry of cells and molecules into brain tissue and eliminates masses formed in the brain by metabolic waste from the cerebral nerve. ECs anchored to each other by tight junctions or adherens junction constitute the most important component of the BBB. Astrocytes and pericytes provide essential support for BBB function with additional contribution from the basement membrane and the glycocalyx (1).

Currently, a lot of studies have shown that vascular endothelial injury is a key pathological process in many vascular diseases, including CSVD (Table 2). In trials, endothelial dysfunction has been shown to be associated with lacunar cerebral infarction (38). An Australian study (39) further demonstrated that in cerebral white matter lesions, the function of ECs and the integrity of BBB were significantly reduced compared to that of normal brain tissue. In addition, the content of intracellular adhesion molecule-1 (ICAM-1) was significantly increased in the diseased region of white matter (40). When focusing on the altered cerebral hemodynamics, it can be found that impaired CBF regulation is widely available in CSVD (41). Furthermore, endothelial impairment is common in the population with atherosclerosis, hypertension, diabetes, and chronic kidney disease (42). Such people are at a higher risk of CSVD. However, the mechanisms has not yet elucidated. In summary, to unfold the nature of CSVD, this review focuses on the relationship between endothelial dysfunction and CSVD.

**Table 2:** Studies suggest that the injury to endothelial cells (ECs) is involved in the pathology of CSVD.

**References**	**Year**	**Conclusion**
Ashby et al. (30)	2021	ECs participate in the CSVD by regulating CBF
Quick et al. (31)	2021	Endothelial dysfunction do damage to BBB and cerebral white matter
Rajani et al. (32)	2018	Endothelial dysfunction is the initial feature of CSVD
Hainsworth et al. (33)	2015	The disruption of BBB caused by endothelial dysfuncion play an important role in the process of CSVD
Nezu et al. (34)	2015	Endothelial dysfunction positively correlates with the severity of WMH and microhemorrhage
Kimura et al. (35)	2012	Anti-endothelial cell antibodies play a role in CSVD
Hassan et al. (36)	2004	Hyperhomocysteinemia, an independent risk factor for CSVD, may play a role by mediating endothelial dysfunction
Leeuw et al. (37)	2002	Endothelial activation is associated with WMH

## Etiologies of and Risk Factors for CSVD and Endothelial Dysfunction

Similar to the cardiovascular risk factors associated with large vessel strokes and heart disease, common systemic vascular risk factors, such as hypertension, diabetes, hyperlipidemia, and hyperhomocysteinemia (43), also play an important role in CSVD. Such factors have been proven to be closely associated with WMH, lacune, and CMB. Among the many risk factors for CSVD, hypertension and age are the most important and independent ones (44). A study showed that EC integrity decreased with age, ultimately leading to an exponential decline in BBB function (45), which might be a potential reason for the high incidence of CSVD among the elderly population. With increasing age, other vascular risk factors further accelerate the development of CSVD (11). Compared to CSVD patients with normal blood pressure, those with hypertension exhibit more severe clinical manifestations and more obvious neuroimaging signs (46). Hypertension may induce microcirculatory change. Rajani et al. (32) also confirmed that EC injury caused by hypertension might be the earliest manifestation of CSVD in rats. Adequate antihypertensive medication contributes to a higher degree of microstructural integrity in cerebral white matter, providing the potential method to delay or prevent the emergence of WMH (47). Unsurprisingly, a recent meta-analysis showed that intensive blood pressure control could prevent the progression of WMH (48). It was found that there is a narrowing of the lumens in the arterioles, which suggested that arteriolosclerosis might be one of the complications associated with hypertension (49). In addition, hyperglycemia and smoking cause CSVD mainly *via* damage to vessel ECs. A study found that type 2 diabetes mellitus (T2DM) increased the risk for WMHs (50). NO produced by ECs plays an important role in blood flow regulation. While the endogenous NO synthase inhibitor, asymmetric dimethylarginine (ADMA), was shown to be significantly elevated in the plasma of patients with CSVD, the level of ADMA correlated with cognitive impairment in patients (51). Both elevated blood glucose levels and smoking (52) can cause vasodilatory dysfunction by downregulating the expression of endothelial nitric oxide synthase (eNOS), which affects endothelium-dependent vasodilation. Furthermore, metabolic syndrome, a combination of abnormalities, including hypertension, T2DM, obesity, and dyslipidemia, is able to stimulate generalized inflammation and promote arteriolosclerosis (53). Insulin resistance (IR) increases the risk of metabolic syndrome. Interestingly, a high triglyceride–glucose (TyG) index (a marker of IR) has been shown to be associated with a higher prevalence and burden of CSVD (54).

As noted previously, the risk factors for atherosclerosis are very similar to those for CSVD. However, almost 90% of cerebral infarctions caused by intracranial atherosclerosis are larger than 2 cm in diameter, and large-artery atherosclerosis occasionally leads to lacunar stroke. In addition, the small perforating arterioles are thickened and stiff, with a reduced lumen size but not containing clots or occlusion. Thus, the pathology of CSVD is distinct from that of atherosclerosis, and the specific mechanisms are still unknown. Furthermore, Arntz et al. (55) conducted a follow-up study of young patients with transient ischemic attack or acute cerebral infarction for nearly 10 years. It was found that these patients developed CSVD 10–20 years earlier than controls and had more severe lesions. Patients with a history of stroke are more susceptible to cerebrovascular diseases because of their poor tolerance to vascular risk factors. Genetic predisposition may be a potential factor, which needs further investigation.

## Vascular EC Injury in CSVD

**Figure 3:**
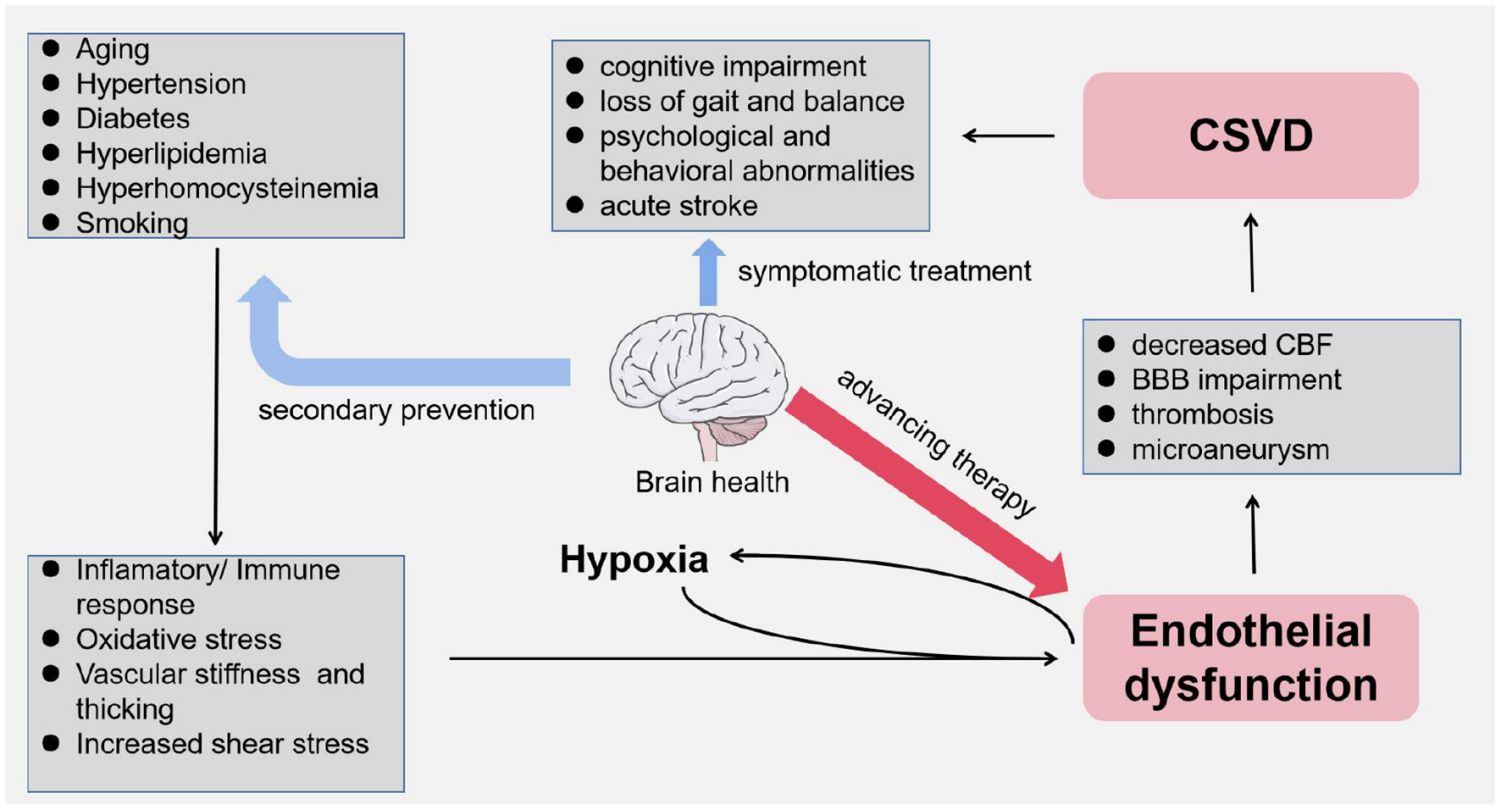
Graphic abstract of this review.

In basilar ganglion, brain stem, centrum semiovale, and subcortical white matter, there are mutiple anastomoses between perforators from pial arteries and intracranial large arteries. The capillary bed composed of these terminal microarteries enables the actual exchange between the blood and the brain, which are diseased under the effect of vascular risk factors as we have discussed. In the 1960s, Fisher (56) performed an autopsy on a patient with lacunar cerebral infarction and first described the pathological features of CSVD in terms of vascular stenosis and hyalinosis, which were mainly found in arteries with a diameter of 40–150 μm. With the breakdown of the integrity of the vascular walls, these smaller arteries have thickened and narrowed (57). Meanwhile, vascular endothelial dysfunction is related to decreased CBF and impaired BBB (58, 59). Endothelial dysfunction is gradually being considered as the driving factor in the development of CSVD (31, 37) (Figure 3).

### CBF Dysregulation in CSVD

Stable CBF ensures a sufficient supply of nutrients and elimination of metabolic waste. Dramatic changes in CBF will result in ischemia or hemorrhage (60), and impaired cerebrovascular hemodynamics is associated with the loss of the structural integrity of cerebral white matter (61).

The neurovascular unit is important in the blood circulation of the brain (4), which provides a basic structure for NVC (Figure 2). In patients with CSVD, an altered adaptive response of the cerebral microvasculature has been found (62). The regulation of CBF depends on intact vascular endothelial structure and function (30). Pericytes have also been shown to play an important role in vasoconstriction. The loss of pericytes severely affects NVC and impairs cerebral vascular autoregulation (63). In summary, the modes of endothelium-derived blood flow regulation include chemical control of CBF, cell–cell interactions, second messenger signaling, and endothelial response to physical forces and inflammatory factors (30). Endothelial injury may alter this adaptation of blood supply to the local energy needs of the brain. In addition, cerebrovascular ECs are sensitive to elevated shear forces and hypoperfusion, which further affect microcirculation regulation due to endothelial dysfunction. This vicious circle aggravates the ischemic–hypoxic brain injury. Anatomically, the deep white matter of the bilateral cerebral hemispheres is supplied by terminal branches of small vessels from two sources, making it extremely fragile to this chronic hypoperfusion. Sustained and intermittent hypoxia causes damage to white matter fiber tracks of the brain resulting in corresponding clinical symptoms, which may be precursors or predictors of dementia (64).

In addition, a few studies have shown reduced CBF in the white matter of patients with CSVD, and the CBF is lower in subjects with more WMH (65, 66). More significantly, researchers confirmed the presence of decreased CBF in the normal-appearing white matter surrounding WMH. This area, termed the CBF penumbra, may be associated with future WMH expansion (67).

### The Function of BBB in CSVD

The blood-brain barrier is made up of ECs, pericytes and astrocytes, which regulates the exchange of substances between the brain and the blood. While ECs tightly regulate this exchange across the BBB (30). In addition to the reduction of CBF, BBB failure also plays an equally important role in the pathological process of CSVD (33). Classical vascular risk factors, salt toxicity, inflammation/infection, and altered hemodynamics can damage the BBB (68).

*Via* dynamic contrast-enhanced MRI, a larger volume with BBB leakage in WMH and cortical gray matter could be found in patients with CSVD (69), and the compromised BBB integrity was associated with total MRI CSVD burden (70). Interestingly, Wong et al. (59) confirmed by functional MRI that BBB permeability was higher in the normal appearing white matter surrounding WMH than in other normal white matter, suggesting that increased BBB permeability might precede the onset of WMH. Researchers also suggested the altered BBB permeability in the normal white matter might be an early indicator of CSVD, which signified a poor prognosis (71, 72). Another study added to mounting evidence that the integrity of BBB was associated with the severity of WMH (39).

White matter hyperintensity pathology might link to the decline of information processing speed (73). Besides, cognitive function descending of patients with CSVD was associated with the degree of BBB leakage at baseline, especially in executive function (74). BBB leakage leads to local microhemorrhage and reduced distal blood flow, which intensifies regional ischemia and hypoxia in the brain. What is more, the leakage and deposition of hematogenous material can lead to perivascular edema (75), which injuries brain cells and leads to demyelination as observed in WMH. Unfortunately, whether BBB breakdown is the starting point of CSVD remains to be studied.

At the same time, it was found that local low CBF was significantly negatively correlated with the permeability of BBB (57). On one hand, it is possible that the low shear stress caused by hypoperfusion leads to the downregulation expression of tight junctions, which results in larger intercellular space and increased material permeation (76). On the other hand, hypoxia may, to some extent, induce the increase of vascular permeability adaptively, allowing more nutrients to enter the brain parenchyma (77). However, the interaction between the two in CSVD and whether they jointly contribute to disease progression remains to be elucidated by further studies.

### Potential Mechanisms of ECs Injury

Endothelial cell injury includes two important pathophysiological processes: EC overactivation and EC dysfunction. Endothelial activation refers to the alterations in the expression, structure, and distribution of endothelial tight junctions responding to a variety of pathological conditions, and ECs turn to an abnormal pro-inflammatory and pro-thrombotic phenotype (78). EC dysfunction is mainly reflected in the imbalance between biomolecules produced by the endothelium that contribute to vasodilation and vasoconstriction, which leads to a series of pathological changes, such as vasoconstriction, leukocyte aggregation, platelet activation, and thrombosis (79). As we have discussed earlier, increased vascular shear stress and ischemic–hypoxic injury both lead to endothelial dysfunction; however, pathological responses, including immune activation, inflammation, and oxidative stress, may be potential mechanisms for EC injury, which ultimately leads to the development of CSVD.

Produced by many different cellular processes, reactive oxygen species (ROS) are the mediators of demyelination and disruption of the BBB. ROS have strong oxidant effects and are able to induce the accumulation and extravasation of leukocytes and trigger the innate immune response. Indeed, several vascular risk factors, such as hypertension, diabetes, smoking, hyperhomocysteinemia, and infections, can promote excess ROS levels. The imbalance between ROS and the antioxidant defense system will cause endothelial injury (80). Homocysteine (HCY) has been shown to promote oxidative injury to the endothelium (81), and total HCY level correlates with CSVD MRI burden (43). In addition, it is believed that smoking-induced oxidative stress can also be a triggering factor that disrupts endothelial integrity (82).

A few experts pointed out that inflammatory factors play an important role in the pathogenesis of CSVD, and patients with ischemic or hemorrhagic lesions on brain MRI have a different distribution of inflammatory markers in their plasma (83). Pro-inflammatory cytokines induce ECs to secrete adhesion molecules and chemokines, recruit immune cells, and generate a waterfall-like inflammatory response, which further impairs the function of endothelium and BBB. In innate immune responses, monocytes activate inflammatory polarization pathways and produce ROS, while macrophages infiltrate the vascular wall, causing smooth muscle cell proliferation and blood vessel remodeling (84). In addition, both cytokines and neopterin secreted by mononuclear macrophages are able to impair BBB by acting directly on the endothelium (85). Neopterin can promote the interaction between EC adhesion molecules and leukocytes, perhaps through the kappa-B pathway (86). In addition, neopterin is able to induce the production of C-reactive protein in the liver, which further generates systemic vascular inflammation (85). In fact, it has been confirmed that adaptive immune responses also participate in the process of endothelial injury. A previous study suggested that aggregated T-cells can attack vascular endothelium directly and decrease CBF (87). Various anti-EC antibodies were detected in the serum of patients with CSVD (35), suggesting that the activation of B-cells might play a role in pathophysiological processes and, to some extent, confirming widespread endothelial dysfunction in CSVD.

## ECs in the Diagnosis and Treatment of CSVD

### Diagnostic Strategies

**Figure 4:**
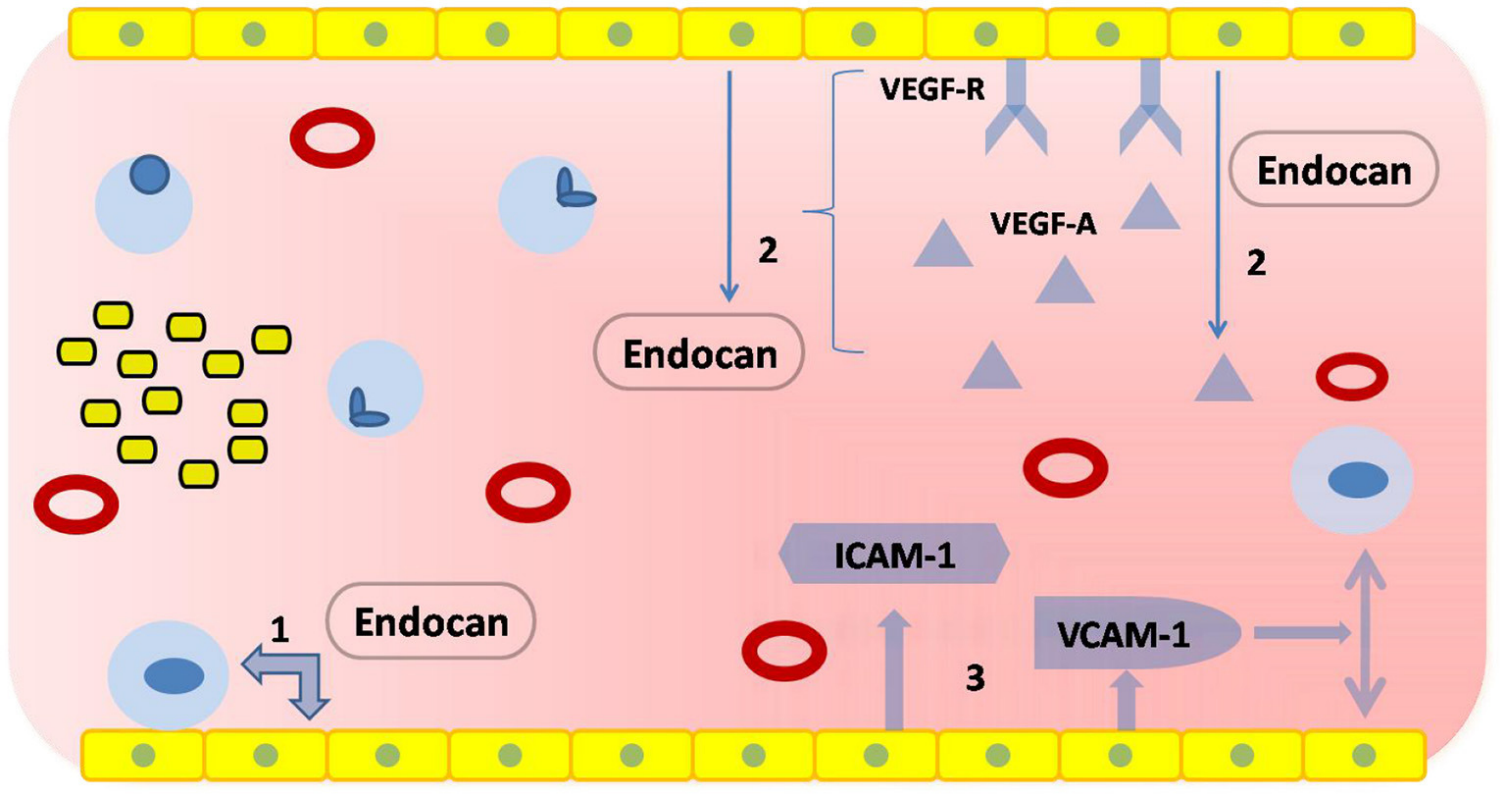
Physiological functions of Endocan. The yellow squares at the upper or lower edges represent ECs, which form vascular endothelium by tight junctions; the red hollow circles represent red blood cells; the light blue circles represent leukocytes; the yellow squares with black borders represent platelets. (1) Endocan regulates the interaction between ECs and leukocytes. (2) Endocan promotes the expression of vascular endothelial growth factor-A (VEGF-A) and enhances the binding of VEGF-A to its receptors. The activation of a VEGF signaling pathway contributes to the production of Endocan. (3) Endocan promotes the release of pro-inflammatory substances.

The autoregulation of cerebral blood flow is the most important feature of cerebral microcirculation. Endothelial injury, the critical part of vascular dysfunction, can be evaluated by flow-mediated dilatation (FMD) of the brachial artery or digital reactive hyperemia index in peripheral arterial tonometry (88, 89). However, these devices have not become popular in clinical practice for various reasons. Thankfully, new techniques, such as digital pulse amplitude tonometry and passive leg movement technique, are on the way (90, 91). In addition, taking BBB leakage into consideration may be helpful in diagnosing CSVD. Another study showed that markers of vascular inflammation and endothelial injury were significantly elevated in blood samples from patients with hypertension-induced CSVD (30). And, it has been found that BBB leakage could be reflected by slow-wave activity during sleep, which could be another biomarker of CSVD (92). However, circulating biomarkers are the ones that provide us with the most opportunities to assess endothelial function (Table 3). These biomolecules are mainly related to endothelial injury and activation (131), including ICAM-1, vascular cell adhesion molecule-1 (VCAM-1), the soluble fraction of von Willebrand factor (vWF), and endothelium-derived exosomes (109, 132). However, most of them are still limited to the laboratory. Previous longitudinal research found that ICAM levels and baseline WMH load were independent predictors of WMH progression (98). Abnormally increased ADMA levels are associated with endothelial dysfunction and the risk of silent brain infarcts (93). Patients with CSVD had higher levels of ADMA in their blood. Matrix metalloproteinase-9 (MMP-9) regulates the metabolism of an extracellular matrix, which is an important component of the blood vessel wall. The levels of MMP-9 were determined to be significantly elevated in patients with WMH (41). In addition, it was confirmed that EC-specific molecule-1 reflected endothelial injury with increased specificity and sensitivity (110). This molecule, also known as Endocan (92), is a soluble dermatopoietin sulfate proteoglycan (DSPG) secreted mainly by ECs. Other studies found that higher levels of Endocan contributed to the production of proinflammatory substances, such as lipopolysaccharide, tumor necrosis factor-α, and interleukins-1β (111). It was hypothesized that Endocan exerted its biological effects through several mechanisms (Figure 4):

①Involve in endothelial activation by regulating the interaction between ECs and leukocytes (133).
②Act on the VEGF signaling pathway to mediate the inflammatory response (134): on one hand, Endocan promotes the expression of VEGF-A and enhances the binding of VEGF-A to its receptors. This process alters vascular permeability; on the other hand, activation of the VEGF signaling pathway contributes to the production of Endocan.
③Promote the release of proinflammatory substances from ECs, including ICAM-1 and VCAM-1 (135).

**Table 3:** Potential biomarkers of endothelial injury.

**Biomolecule**	**Function**	**Test**	**Variation**	**Conclusion**	**References**
asymmetric dimethylarginine (ADMA)	NO synthase inhibitor	ELISA	↑	causing endothelial dysfunction by blocking the activity of endogenous NO synthase	(93, 94)
VCAM-1	inducing the interactions between leukocytes and endothelium	ELISA	↑	higher expression of VCAM-1 is related with endothelial activation	(82, 95, 96)
ICAM-1	inducing the interactions between leukocytes and endothelium; participating in the substance transmembrane transportation	ELISA	↑	higher expression of ICAM-1 is related with endothelial activation	(82, 97–99)
myeloperoxidase (MPO)	involved in the impairment of tissues and inflammation	ELISA	↑	MPO can cause endothelil injury and dysfunction	(100)
Claudin-5	maintaining the structure of tight junction	ELISA	↑	Decreased Claudin-5 level are associated with the disruption of BBB integrity	(101, 102)
matrix metalloproteinase-2/9 (MMP-2/MMP-9)	degrading the components of extracellular matrix	gelatin zymography	↑	Increased MMP-2/MMP-9 level are associated with the disruption of BBB integrity	(103–105)
endothelin-1 (ET-1)	regulating vasoconstriction	ELISA	↑	Excess ET-1 causes pathological vasoconstriction	(106)
vWF	facilitating clotting and the adhesion of platelets	ELISA;gelatin zymography;Immunoelectrophoresis	↑	Injured ECs release polymeric vWF, further causing vascular dysfunction	(106–108)
endothelial microparticles (EMPs)	involved in the intercellular communication	flow cytometry; atomic force microscope; electron microscope	↑	Activated ECs release specific EMPs into the bloodstream	(92, 93, 98, 109–117)
Endoglin (CD105)	involved in the angiogenesis, vasodilation, and inflammation	ELISA; Western blot	↑	Increased Endoglin worsen inflammation and weak the relaxation response of the vessel	(118–123)
Endocan (ESM-1)	inducing the interactions between leukocytes and endothelium; regulating the vascular function	ELISA	↑	Injured ECs release Endocan into the bloodstream, which promotes the infiltrating of leucocytes	(124–127)
micRNA	participating in various kinds of endothelial function	PCR	↑/↓	Several micRNAs expressed specifically by ECs can suggest the endothelial dysfunction	(128–130)

*↑:increased expression level; ↓:decreased expression level*.

In contrast to blood biomarkers, there are a few studies assessing biomarkers of CSVD or endothelial dysfunction using cerebrospinal fluid (CSF) samples. Elevated albumin and other serum proteins have been found in the CSF of patients with VD (42). An increased albumin CSF/serum ratio, though not specific, may be useful in reflecting BBB dysfunction. In addition, the CSF level of ICAM-1 and VCAM-1 were higher in diabetics with cerebral vascular injury (136), but the correlation between adhesion molecules in CSF and CSVD is still unknown. Further studies are needed to search for CSF biomarkers of CSVD.

Currently, several methodological issues prevent clinical practice, and the replication of these results is indispensable. On one hand, the distribution of biomolecules in different populations is also with heterogeneity and complexity, and the variability of labs or measuring approaches will induce a great measurement error. On the other hand, changes in peripheral blood biomarkers may not be able to fully reflect the degree of cerebral tissue damage and the state of brain function, and the content of biological molecules may be influenced by various physiological or pathological conditions. In addition, it may be more reliable to assess endothelial function by monitoring changes of a set of molecules rather than a single molecule.

### Therapeutic Directions

The goal of treating CSVD is to prevent stroke, delay cognitive decline, improve gait, and resolve psychological abnormalities. According to the lesion changes revealed by MRI, doctors can observe the curative effect (137). Currently, clinical interventions for CSVD are mainly limited to health management of risk factors. Tight blood pressure control may be beneficial in preventing or delaying the onset of CSVD. It is important to note that blood pressure variability (BPV) comes to be valued in treatment. In addition, low-dose rosuvastatin (a kind of lipid lower agents) may be a reasonable therapy for CSVD (138). In a recent study, the results suggested that nimodipine (a kind of antihypertensive medications) combined with rosuvastatin was safe and effective in treating mild cognitive impairment in patients with CSVD (139). Apart from the routine drug therapy, healthy lifestyles, such as smoking cessation, low-salt diet, and exercise, may help to halt or delay the progression of CSVD. Antiplatelet therapy is one of the most important measures in the treatment of cerebrovascular disorders. Due to hemodynamic fluctuations in the cerebral microcirculation and impaired BBB, patients with CSVD have a higher risk of bleeding during the application of antithrombotic drugs, especially in patients with more microhemorrhage foci (140). Something else we need to be careful about is the higher prevalence of aspirin resistance in patients with CSVD (141). Unfortunately, donepezil and memantine, which are commonly used to improve cognitive function, have little effect on the cognitive impairment caused by CSVD.

Endothelial dysfunction, the keystone of this article, is a key in the pathological process of CSVD. Therefore, the treatment of ECs is expected to be a breakthrough. The concept of “endothelial therapy” was proposed in the late 1990s. The therapy was aimed to prevent and repair EC injury and was mainly involved in the treatment of cardiovascular diseases (142). A study (32) has confirmed that reversal of endothelial dysfunction could reduce the cerebral white matter damage in CSVD rats, providing a basic theory for subsequent clinical applications.

Firstly, a healthy lifestyle may be beneficial to endothelial health. A study found that the Mediterranean diet could modulate endothelial function, even in those with severe endothelial dysfunction (143). In addition, aerobic exercise training was considered to provide the same benefit (144). Secondly, existing drugs may be effective in protecting ECs. For example, it has been found that metformin may exert protective effects in preventing endothelial dysfunction (106). Carvedilol, a nonselective beta- and alpha-receptor antagonist, was found to have the antioxidative potential *in vitro* (145). Anti-inflammation and anti-oxidation agents may play a positive role in endothelial health. However, more randomized controlled trials and experimental studies are needed to confirm the above conclusions, and whether these interventions are effective in delaying the progression of CSVD needs to be further clarified. What is more, new therapeutic strategies targeting endothelial repair are worth investigating. Several cytokines or molecules may be useful in endothelial repair. Scholars have found that granulocyte colonystimulating factor (G-CSF) had protective effects on endothelial impairment and WM injury in CSVD. G-CSF promoted the expression of VEGF and downregulated the level of MMP-9, thus repairing the cerebral vascular endothelium (146). Endothelial progenitor cells (EPCs) are capable of repairing injured endothelium, thus providing promising therapy to treat CSVD (147). In addition, several plant extracts have also been shown to be potential for alleviating the EC injury (148, 149). The pharmacological functions of these natural substances mainly include lessening oxidative injury, decreasing EC apoptosis, and reducing the inflammation response. In addition, the therapeutic potential of endothelium-specific microRNAs for the treatment of EC dysfunction is attracting attention (150), and antibodies against the endothelium may be a target for immunotherapy in the future.

## Conclusion

This review focuses on the function of ECs, particularly their pathological changes in the process of CSVD. We hold the opinion that ECs are culprits and victims during CSVD at the same time. Increased shear stress or hypoxia causes EC dysfunction. More importantly, endothelial activation enhances the inflammatory response and immune reaction, leading to BBB leakage and impaired cerebral blood supply. In addition, reduced endothelial NO synthesis and the pro-thrombotic state exacerbate the ischemic brain damage.

In summary, it is important to continue to deepen our knowledge of endothelial dysfunction to understand the nature of CSVD. Identification of endothelial-specific markers will be useful for both laboratory studies and clinical trials. And, it certainly makes sense to therapeutically target ECs during CSVD.

## Author Contributions

TB reviewed the literature and designed and drafted this manuscript. SY revised the manuscript. JF was involved in the design and revision of the manuscript. All authors contributed to this article and approved the submitted version.

## Funding

The study was financially supported by grants from the National Natural Science Foundation of China (81771271) and the Outstanding Scientific Fund of Shengjing Hospital (M0475) awarded to JF.

## Conflict of Interest

The authors declare that the research was conducted in the absence of any commercial or financial relationships that could be construed as a potential conflict of interest.

## Publisher's Note

All claims expressed in this article are solely those of the authors and do not necessarily represent those of their affiliated organizations, or those of the publisher, the editors and the reviewers. Any product that may be evaluated in this article, or claim that may be made by its manufacturer, is not guaranteed or endorsed by the publisher.
